# Supplementary material for: Using slisemap to interpret physical data
Source: PLoS One. 2024 Jan 25;19(1):e0297714. doi: 10.1371/journal.pone.0297714 (PMC10810528; doi:10.1371/journal.pone.0297714)
Supplement: S1 File — (PDF) [file pone.0297714.s001.pdf]

# Supporting information

## S1 Chosen explainable features for GeckoQ

The following variables available from the GeckoQ data were utilized as *Explainable features*: Number of atoms, number of carbon atoms, C=C (non-aromatic, hydroxyl (alkyl), aldehyde, ketone, carboxylic acid, ester, ether (alicyclic), nitrate, nitro, carbonylperoxynitrate, peroxide, hydroperoxide, carbonylperoxyacid. The functional groups 'C=C-C=O in non-aromatic ring', 'aromatic hydroxyl', 'nitroester' were also available. Still, we decided to drop them because fewer than 2 % of the data had an entry for these properties.

## S2 Incidence of functional groups by cluster in GeckoQ

See Fig. S1 for the incidence of functional groups for each of the clusters described in Section 4.

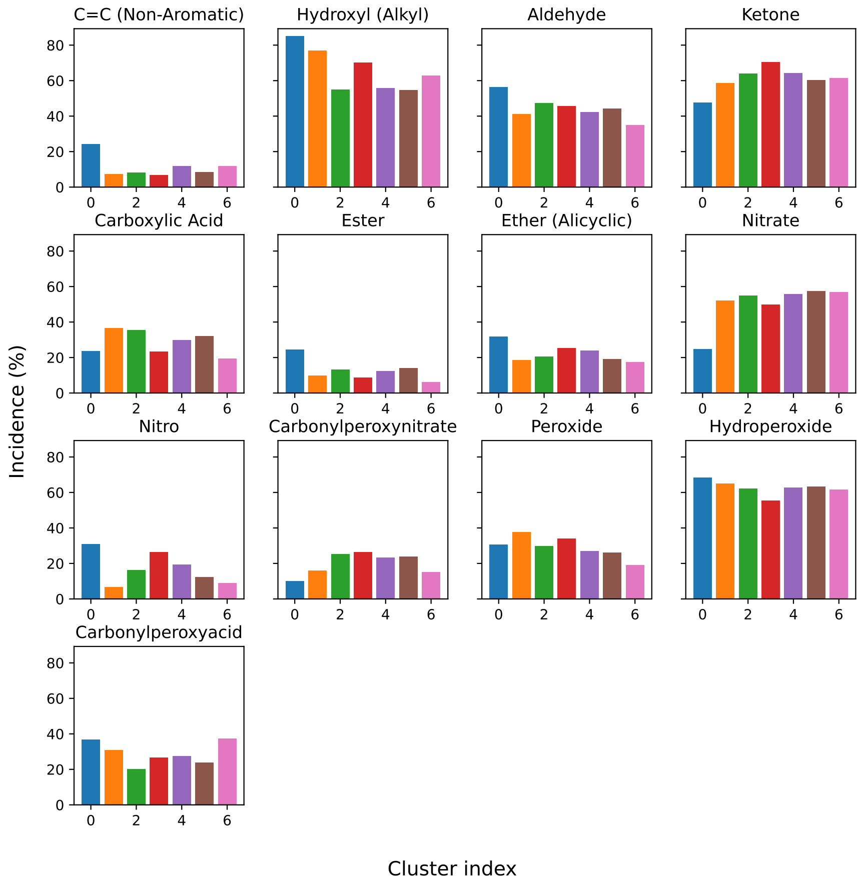


**Fig S1.** Functional groups by cluster.

## S3 Explanation metrics

*Local loss.* The local loss (called "fidelity" in [8]) measures how well the local models learned by SLISEMAP predict the target. Mathematically, local loss is expressed as

$$\frac{1}{n}\sum_{i=1}^{n} l(f_i(\mathbf{x}_i), \mathbf{y}_i),$$

where $f_i$ are the local models and $l$ is the loss function as defined in Sect. 3.1. We are not only interested on the performance of the individual local model-target pairs but also how well the local models can predict the neighbouring points in the embedding. To find the neighbours, we use the $k$ nearest neighbours (in the embedding when an embedding is produced; if not, in the original data space), rendering

$$\frac{1}{n}\sum_{i=1}^{n}\frac{1}{k}\sum_{j\in \text{k-NN}(i)} l(f_i(\mathbf{x}_j), \mathbf{y}_j).$$

In this paper, we chose to use $k = \lfloor 0.1N \rfloor$ where $N$ is the number of data items. A smaller value of local loss is better.

*Coverage.* Coverage [7] measures how well local models generalise to other data points. It can be calculated by counting the number of data items that have a local loss less than a set threshold $l_0$:

$$\frac{1}{n}\sum_{i=1}^{n}\frac{1}{n}\sum_{j=1}^{n} |\{j : l(g_i(\mathbf{x}_j), \mathbf{y}_j) < l_0\}|.$$

We are also interested in the local coverage of the models. Thus, we can limit the coverage testing to the $k$ nearest neighbours as above and with the same value of $k$. We get

$$\frac{1}{n}\sum_{i=1}^{n}\frac{1}{k}\sum_{j\in \text{k-NN}(i)} |\{j : l(g_i(\mathbf{x}_j), \mathbf{y}_j) < l_0\}|.$$

In this paper, we have chosen the loss threshold to be the 0.3 quantile of the losses of a global linear model (similar to [8]). A higher coverage indicates better performance.
